# 795 Autologous skin cell suspension application for toxic epidermal necrolysis: a case report

**DOI:** 10.1093/jbcr/irac012.345

**Published:** 2022-03-23

**Authors:** Alan Pang, Theophilus Pham, Sarah Felan, Akshay Raghuram, Michelle Terry, Deepak Bharadia, John A Griswold

**Affiliations:** Texas Tech University Health Science Center, Lubbock, Texas; Texas Tech University Health Sciences Center, Lubbock, Texas; Texas Tech University Health Sciences Center, Lubbock, Texas; Texas Tech University Health Sciences Center, Lubbock, Texas; Texas Tech University Health and Science Center, Lubbock, Texas; Texas Tech University Health Sciences Center, Lubbock, Texas; Texas Tech University Health Sciences Center, Lubbock, Texas

## Abstract

**Introduction:**

Toxic epidermal necrolysis (TEN) is a drug-mediated disease which occurs at the same level of a partial thickness thermal injury. It has been treated with frequent dressing changes and supportive care as there are no definitive therapies. The pathophysiology involves necrosis of keratinocytes at the dermal-epidermal junction leading to sloughing of the epidermis and sub-epidermal bullae. Patients have massive fluid and electrolyte dysregulation due to chronic wounds that are susceptible to infection. This case report describes the application of autologous skin cell suspension (ASCS) to a patient with TEN. His hospital course included arrest of the disease process, timely closure of the wound, and minimal wound care. To our knowledge, this is the first case of TEN treated with ASCS.

**Methods:**

The patient was mechanically debrided with a water scalpel hydrosurgery system to remove all devitalized tissue. A donor graft for ASCS was then obtained at standard depth from unaffected skin at the ankle and was processed in the standard fashion and applied to the wound. Clear plastic dressing with a non-adherent, antibiotic impregnated cloth dressing followed by dry non-shear dressing were then applied to the torso. Extremities were dressed in clear plastic dressing, antibiotic impregnated cloth dressing, zinc paste impregnated cloth, dry gauze roll, and elastic compression bandage. This was performed over the patient’s anterior surface and subsequently on the posterior one week later. Dressings were taken down on post-operative day (POD) 6 and replaced as appropriate.

**Results:**

The patient demonstrated 60% closure of wounds on his anterior torso at POD 6. By POD 8, the patient's anterior torso and face were completely closed. His second application of ASCS to his posterior closed in a similar timeline. The patient was discharged on POD 16 from his initial intervention with 2% total body surface area open. At POD 30, his wounds were healed with the return of a large portion of pigmentation.

**Conclusions:**

TEN is a rare, high-mortality disease without efficacious therapy. Given that the wounds are at a similar level to a partial thickness burn, ASCS could be a new intervention which could mitigate open wounds, frequent dressing changes, prolonged hospital stays, and mortality.

